# Current Role of Immunotherapy in Gastric, Esophageal and Gastro-Esophageal Junction Cancers—A Report from the Western Canadian Gastrointestinal Cancer Consensus Conference

**DOI:** 10.3390/curroncol29050257

**Published:** 2022-04-29

**Authors:** Karen Mulder, Howard Lim, Deepti Ravi, Shahida Ahmed, Bryan Brunet, Janine Davies, Corinne Doll, Dorie-Anna Dueck, Vallerie Gordon, Pamela Hebbard, Christina A. Kim, Duc Le, Richard Lee-Ying, John Paul McGhie, Jason Park, Daniel J. Renouf, Devin Schellenberg, Ralph P. W. Wong, Adnan Zaidi, Shahid Ahmed

**Affiliations:** 1 Cross Cancer Institute, Alberta Health Services, Edmonton, AB T6G 1Z2, Canada; 2 British Columbia Cancer Agency, Vancouver, BC V5Z 4E6, Canada; hlim@bccancer.bc.ca (H.L.); jan.davies@bccancer.bc.ca (J.D.); drenouf@bccancer.bc.ca (D.J.R.); 3 Saskatchewan Health Authority, Saskatoon, SK S7K 0M7, Canada; deepti.ravi@saskhealthauthority.ca; 4 CancerCare Manitoba, Winnipeg, MB R3E 0V9, Canada; sahmed1@cancercare.mb.ca (S.A.); vallerie.gordon@cancercare.mb.ca (V.G.); pamela.hebbard@cancercare.mb.ca (P.H.); ckim3@cancercare.mb.ca (C.A.K.); jpark@sbgh.mb.ca (J.P.); ralph.wong@cancercare.mb.ca (R.P.W.W.); 5 Saskatoon Cancer Center, Saskatchewan Cancer Agency, Saskatoon, SK S7N4H4, Canada; bryan.brunet@saskcancer.ca (B.B.); dorie-anna.dueck@saskcancer.ca (D.-A.D.); duc.le@saskcancer.ca (D.L.); adnan.zaidi@saskcancer.ca (A.Z.); 6 Arnie Charbonneau Cancer Institute, Alberta Health Service, Calgary, AB T2N 4Z6, Canada; corinne.doll@albertahealthservices.ca (C.D.); richard.lee-ying@ahs.ca (R.L.-Y.); 7 British Columbia Cancer Agency, Victoria, BC V8R 6V5, Canada; jmcghie@bccancer.bc.ca; 8 British Columbia Cancer Agency, Surrey, BC V3V 1Z2, Canada; dschellenberg@bccancer.bc.ca

**Keywords:** gastroesophageal cancer, stomach cancer, esophageal cancer, gastroesophageal junction cancer, immunotherapy, checkpoint inhibitors

## Abstract

Gastric, esophageal and gastro-esophageal junction cancers are associated with inferior outcomes. For early-stage disease, perioperative chemotherapy or chemoradiation followed by surgery is the standard treatment. For most patients with advanced upper gastrointestinal tract cancers, platinum-based chemotherapy remains a standard treatment. Recently, several randomized clinical trials have demonstrated the benefit of immunotherapy involving checkpoint inhibitors alone or in combination with chemotherapy in patients with gastro-esophageal cancer and have changed the treatment landscape. The Western Canadian Gastrointestinal Cancer Consensus Conference (WCGCCC), involving experts from four Western Canadian provinces, convened virtually on 16 June 2021 and developed the recommendations on the role of immunotherapy in patients with gastro-esophageal cancer.

## 1. Term of References

### 1.1. Purpose

The Western Canadian Gastrointestinal Cancer Consensus Conference (WCGCCC) aims to develop a consensus opinion of health care professionals from four Western Canadian provinces (British Columbia, Alberta, Saskatchewan and Manitoba), attempting to define best care practices and to improve care and outcomes for patients with gastrointestinal cancers.

### 1.2. Participants

The WCGCCC welcomes medical oncologists, radiation oncologists, surgical oncologists, pathologists, radiologists, gastroenterologists and allied health professionals from Western Canada who are involved in the care of patients with gastrointestinal malignancies (Table S1).

### 1.3. Target Audience

The recommendations presented here are targeted at health care professionals involved in the care of patients with gastric, esophageal and gastro-esophageal junction cancers.

### 1.4. Basis of Recommendations

The recommendations are based on the presentation and discussion of the best available evidence at the time of the meeting.

## 2. Consensus Question

### 2.1. What Is the Role of Immunotherapy in Gastric, Esophageal and Gastroesophageal Junction (GEJ) Cancers Both in the Metastatic and Adjuvant Setting?

Patients should have mismatch repair (MMR) and PD-L1 combined positive score (CPS) testing for decision making of immunotherapy. The CPS assay should be performed by a pathologist with experience with this assay.

#### 2.1.1. Early-Stage Disease

Patients with resected esophageal or GEJ cancer who have received neoadjuvant chemoradiotherapy and have residual disease should receive 1 year of adjuvant nivolumab.

#### 2.1.2. Advanced-Stage HER2-Negative Disease

First-line therapy with fluoropyrimidine/platinum and a checkpoint inhibitor should be considered in metastatic or unresectable esophageal, gastric and GEJ cancers. In the setting of adenocarcinoma with a PD-L1 score of <1%, a checkpoint inhibitor has not been shown to be beneficial in the first-line setting.

In patients where chemotherapy may not be an option, immunotherapy could be considered in PD-L1 positive tumors.

In patients who have not received immunotherapy as first-line treatment, immunotherapy could be considered in PD-L1 positive tumors.

In patients with deficient MMR (dMMR) tumors, immunotherapy should be considered.

## 3. Introduction

Upper gastrointestinal malignancies including cancers of the stomach, esophagus and gastro-esophageal junction (GEJ) are globally common and have been associated with a high mortality-to-incidence ratio [1,2,3]. Surgery is the primary treatment for the localized disease; however, many patients develop recurrence after curative surgery. Multi-modality therapy involving radiation and/or chemotherapy is the current standard of care for high-risk disease to reduce the risk of recurrence. Patients with early-stage esophageal and GEJ cancers are treated with preoperative chemoradiation therapy or perioperative multi-agent chemotherapy followed by surgery, whereas patients with stomach cancer are primarily treated with perioperative chemotherapy [4,5,6]. Until recently, for most patients with advanced-stage HER2 negative disease, platinum-based chemotherapy remained the standard therapy [7,8]. The advent of checkpoint inhibitors has revolutionized the management of many solid organ cancers [9,10,11,12]. Emerging data have demonstrated their efficacy in upper gastrointestinal cancer, including advanced esophageal, gastric and GEJ cancers and, more recently, in early-stage esophageal and GEJ cancers [13,14,15,16,17,18]. The WCGCCC reviewed the existing evidence of immunotherapy in patients with gastric, esophageal and gastroesophageal cancers and developed recommendations regarding the indication of immunotherapy in such patients.

## 4. Methods

The consensus question was developed by the WCGCCC Executive Committee. The Executive Committee members KM and SA co-chaired the virtual consensus meeting. HL and DR presented the evidence of the efficacy of immunotherapy in upper gastrointestinal cancers and related biomarker testing. Each provincial group independently reviewed the evidence and developed a consensus statement. The four provincial groups subsequently met together, reviewed all four consensus statements and developed a final consensus statement agreed upon by the conference participants.

## 5. Results: Summary of Evidence

### 5.1. Predictive Biomarkers

The accumulating evidence suggests the benefit of immunotherapy both in patients with early-stage esophageal and GEJ cancers and in patients with advanced gastric, esophageal and GEJ cancers [13,14,15,16,17,18]. Predictive biomarkers, however, are important to identify patients who will benefit from checkpoint inhibitors.

#### 5.1.1. MMR Genes

MMR genes include *MSH2, MSH3, MSH6, MLH1, MLH3, PMS1* and *PMS2*, which are involved in the repair of mismatched bases [19,20]. A defect in DNA mismatch repair results in a microsatellite instability (MSI) phenotype [19,20]. Microsatellites or short tandem repeats are comprised of repeated sequences of 1–6 nucleotides [21]. MSI reflects genetic instability in the underlying cancer and is classified as a high microsatellite instability (MSI-high or MSI-H), microsatellite stability (MSI-stable) or low microsatellite instability (MSI-low) tumor [22]. An increased mutational burden and the excess of neoantigens due to an impaired ability to repair DNA damage in dMMR or MSI-high cancers have been associated with a positive response to immunotherapy in solid organ cancers, including esophageal, gastric and GEJ cancers [9,10,11,12]. Immunohistochemistry (IHC) is an easy and quick test to determine the loss of one or more MMR proteins and aberrations in MMR genes. Alternatively, molecular tests that include next-generation sequencing are effective assays to determine defective MMR genes [19]. About 3–7% of patients with advanced gastroesophageal adenocarcinomas have MSI-H tumors, and most of these cancers are positive for PD-L1 [23,24].

#### 5.1.2. Programmed Death-Ligand 1 (PD-L1) and the Combined Positive Score (CPS)

PD-L1 is expressed in many tissues, including tumor cells. Its interaction with the PD-1 receptor in immune cells results in the inactivation of T cells, resulting in downregulation of the immune response and promotion of self-tolerance [25]. PD-L1 testing has predictive value in many cancers for identifying patients who may benefit from an immune checkpoint inhibitor [18,26]. PD-L1 expression is an important mechanism for immune evasion by the cancer cells that facilitates their proliferation, progression and metastases [27]. PD-L1 expression in upper gastrointestinal tract adenocarcinomas can be detected on formalin-fixed paraffin-embedded tissue sections. A qualitative IHC assay with mouse monoclonal antibodies is utilized, and the CPS on tissue slide sections is calculated [28]. The CPS is essentially the number of PD-L1-positive cells (tumor and inflammatory cells (i.e., lymphocytes and macrophages)) divided by the total number of viable tumor cells and multiplied by 100.

PD-L1 expression exists along a continuum, and the thresholds to separate positive PD-L1 expression from negative PD-L1 expression remain under debate. A specimen is considered PD-L1 expression positive if CPS is ≥1; however, clinical utility thresholds vary depending on the type of tumor and the antibody clone used. A calculated CPS can be over 100; however, the maximum score that can be reported is 100. The type of clone is also important to consider. For instance, the PD-L1 IHC 22C3 clone is intended for use in detecting the PD-L1 protein in gastric or GEJ adenocarcinomas in patients who could potentially be treated with pembrolizumab. Most modern clinical trials have used a pre-specified CPS threshold for their primary endpoints. Some trials have also used tumor proportion score (TPS), the percentage of viable tumor cells showing partial or complete membrane staining of PD-L1 at any intensity. The use of a higher threshold of CPS ≥ 5 or ≥10 compared to CPS ≥ 1 has resulted in increasing the number of patients who will likely benefit from immunotherapy [29].

##### Evaluation of Assay and Test

It is recommended that all assessments be performed by trained pathologists and correlated with the patient’s clinical and imaging findings. At least 100 viable tumor cells are needed for adequate assessment. If enough tissue is not available, a deeper level of block can be attempted. Otherwise, another block can be utilized. If a patient’s specimens include ≥1 biopsy on a slide, all the tissue on the slide needs to be evaluated.

When assessing the IHC, any convincing partial/complete linear membranous staining at 20× magnification (score ≥ 1) of viable tumor cells, which is distinct from cytoplasmic staining, is considered PD-L1 staining and should be included in the scoring. In addition to tumor cells, inflammatory cells are scored as long as they are directly associated with the response against the tumor being scored.

Cells excluded in the scoring include tissue with poor morphology, necrotic tissue, nonspecific or excess background staining, non-staining tumor cells, tumor cells with cytoplasmic staining only and areas showing dysplasia only (with no invasion).

It is recommended that all patients with newly diagnosed unresectable locally advanced or metastatic esophageal, gastric and GEJ cancers have their tumors examined for MSI-H/dMMR status and overexpression of PD-L1 using the CPS.

### 5.2. Immunotherapy in Patients with Early-Stage Esophageal or GEJ Cancer

#### Adjuvant Treatment in Resected Esophageal or GEJ Cancer Post-Chemoradiation

The standard treatment options for patients with localized esophageal or GEJ cancer are either preoperative chemoradiation therapy or perioperative chemotherapy [4,5,6].

Checkmate 577 is a phase III randomized, double-blind, placebo-controlled trial evaluating nivolumab at a dose of 240 mg every 2 weeks for 16 weeks, then changed to 480 mg every 4 weeks versus placebo (2:1), for a maximum duration of 1 year [14]. Patients with resected stage II or stage III esophageal or GEJ cancer and residual pathological disease post-neoadjuvant chemoradiotherapy were enrolled. Overall, 532 patients received nivolumab versus 262 patients who received placebo. The median disease-free survival was 22.4 months with nivolumab compared with 11.0 months with placebo (HR 0.69 for disease recurrence or death, *p* = 0.001) [Table 1]. On post hoc analysis, disease-free survival benefit of adjuvant nivolumab was demonstrated in patients with PD-L1 positive and PD-L1 negative tumors. Based on the results, 1 year of adjuvant nivolumab is recommended in patients with localized esophageal or GEJ cancer with residual disease following preoperative chemoradiation therapy regardless of PD-L1 status.

### 5.3. Immunotherapy for Metastatic or Locally Advanced Unresectable Gastric, Esophageal and GEJ Cancers

Until recently, platinum-based chemotherapy was the standard of care for patients with previously untreated advanced gastroesophageal squamous cell cancer and HER2-negative adenocarcinoma. Several randomized clinical trials have demonstrated the benefit of immunotherapy involving checkpoint inhibitors alone or in combination with chemotherapy or a CTLA-4 inhibitor in these patients and has changed the treatment landscape of advanced gastroesophageal cancer (Table 1).

#### 5.3.1. Immunotherapy for Previously Untreated Patients or First-Line Therapy

##### Trials Using Checkpoint Inhibitors Alone or in Combination with Chemotherapy

In the Keynote-062 trial, 763 patients with untreated advanced gastric or GEJ cancer with a PD-L1 CPS ≥ 1 were randomized 1:1:1 to pembrolizumab alone, pembrolizumab plus chemotherapy (cisplatin plus 5-fluorouracil or capecitabine) or chemotherapy plus placebo [16]. At the median follow-up period of 29.4 months, in patients with tumor CPS ≥ 1, pembrolizumab alone was associated with a median OS of 10.6 months compared to 11.1 months with chemotherapy alone (HR, 0.91; 99.2% CI, 0.69–1.18). In patients with tumor CPS ≥ 10, pembrolizumab alone was associated with a median OS of 17.4 months compared to 10.8 months with chemotherapy alone (HR, 0.69; 95% CI, 0.49–0.97). In patients with a CPS ≥ 1, pembrolizumab plus chemotherapy resulted in a median OS of 12.5 months vs. 11.1 months with chemotherapy alone (HR, 0.85; 95% CI, 0.70–1.03). Likewise, in patients with a CPS ≥ 10, pembrolizumab plus chemotherapy resulted in a median OS of 12.3 months vs. 10.8 months with chemotherapy alone (HR, 0.85; 95% CI, 0.62–1.17).

A post hoc analysis of three trials that included KEYNOTE-062 involving patients with MSI-H gastric or GEJ cancer showed that the median OS of patients who received pembrolizumab was not reached, and the median progression-free survival (PFS) varied from 17.8 months to not reached in the first and subsequent lines [23].

##### Trials Using Checkpoint Inhibitors in Combination with Chemotherapy or a Cytotoxic T-Lymphocyte-Associated Protein 4 (CTLA-4) Inhibitor

Checkmate 649 is a phase III trial that enrolled 1581 adults with previously untreated, unresectable, non-HER2-positive gastric, esophageal and GEJ adenocarcinoma, regardless of PD-L1 expression [15]. During enrollment, the population was amended to patients whose tumors had a PD-L1 CPS ≥ 5 based on the Checkmate 032 results of the gastroesophageal cohort. PD-L1 expression was similar to the Checkmate 648 trial. Patients were randomized arms to receive nivolumab (360 mg every 3 weeks or 240 mg every 2 weeks) and chemotherapy (XELOX every 3 weeks or FOLFOX every 2 weeks), nivolumab and ipilimumab (a CTLA-4 inhibitor) or chemotherapy alone. The results of the nivolumab and ipilimumab arm will be reported later and remains blinded at the time of this report. The median follow-up for OS was as follows: nivolumab plus chemotherapy, 13.1 months (IQR, 6.7–19.1), and chemotherapy, 11.1 months (IQR, 5.8–16.1). Nivolumab plus chemotherapy resulted in improvements in OS in tumors with a CPS ≥ 5 (14.4 months vs. 11.1 months; HR, 0.71; 98.4% CI, 0.59–0.86; *p* < 0.0001), a CPS ≥ 1 (14.0 months vs. 11.3 months; HR, 0.77; 99.3% CI, 0.64–0.92; *p* < 0.0001) and in all randomized patients (13.8 months vs. 11.6 months; HR, 0.80; 99.3% CI, 0.68–0.94; *p* = 0.0002). The HRs for OS with nivolumab plus chemotherapy versus chemotherapy in patients with a PD-L1 CPS < 1 and <5 were 0.92 (95% CI, 0.70–1.23) and 0.94 (95% CI, 0.78–1.13), respectively.

In the Keynote-590 trial, 749 patients with untreated advanced esophageal or Siewert type 1 GEJ adenocarcinoma or squamous cell cancer, irrespective of tumor PD-L1 status, were randomized to pembrolizumab or 5-fluorouracil and cisplatin plus placebo for up two years of treatment [17]. At the median follow-up period of 22.6 months, in patients with esophageal squamous cell carcinoma (ESCC) and PD-L1 CPS ≥ 10, pembrolizumab plus chemotherapy was associated with a median OS of 13.9 months compared to 8.8 months with chemotherapy alone (HR, 0.57; 95% CI, 0.43–0.75). In patients with ESCC, pembrolizumab plus chemotherapy was associated with a median OS 12.6 months vs. 9.8 months with chemotherapy alone (HR, 0.72; 95% CI, 0.60–0.88). In all randomized patients, pembrolizumab plus chemotherapy resulted in a median OS of 12.4 months vs. 9.8 months with chemotherapy alone (HR, 0.73; 95% CI, 0.62–0.86). Pembrolizumab plus chemotherapy compared to chemotherapy alone was associated with better PFS in patients with ESCC (6.3 months vs. 5.8 months; HR, 0.65; 95% CI, 0.54–0.78), in patients with tumor PD-L1 CPS ≥ 10 (7.5 months vs. 5.5 months; HR, 0.51; 95% CI, 0.41–0.65; *p* < 0.0001) and in all randomized patients (6.3 months vs. 5.8 months; HR, 0.65; 95% CI, 0.55–0.76; *p* < 0.0001).

CheckMate 648 is a phase III trial that enrolled 970 patients with untreated, unresectable advanced, recurrent or metastatic esophageal squamous cell cancer, regardless of tumor cell PD-L1 expression. PD-L1 immunohistochemistry was determined using the Dako PD-L1 28–8 pharmDx assay. A total of 970 patients were randomized to nivolumab (240 mg Q2W) plus chemotherapy (fluorouracil plus cisplatin Q4W), nivolumab (3 mg/kg Q2W) plus ipilimumab (1 mg/kg Q6W) or chemotherapy alone [13]. With 13 months follow-up, nivolumab plus chemotherapy and nivolumab plus ipilimumab led to statistically significant improvements in OS versus chemotherapy in patients with tumor cell PD-L1 ≥ 1%—15.4 months versus 13.7 months versus 9.1 months (HR, 0.54; 99.5% CI, 0.37–0.80; *p* < 0.0001 and HR, 0.64; 98.6% CI, 0.46–0.90; *p* = 0.001, respectively). The median survival of all randomized patients was 13.2 months versus 12.8 months versus 10.7 months (HR, 0.74; 99.1% CI, 0.58–0.96; *p* = 0.0021 and HR, 0.78; 98.2% CI, 0.62–0.98; *p* = 0.01, respectively).

In the ATTRACTION-4 trial, 742 Asian patients were randomized to nivolumab plus chemotherapy (S-1 plus oxaliplatin or capecitabine plus oxaliplatin) or placebo plus chemotherapy [30]. The combination of immuno- and chemotherapy was associated with a significantly superior PFS of 10.5 months versus 8.3 months with chemotherapy alone (HR, 0.68; 98.51% CI, 0.51–0.90; *p* = 0.0007), meeting the primary endpoint. However, at the median follow-up period of 26.6 months, there was no statistically significant difference in median OS (17.5 months vs. 17.2 months; HR, 0.90; 95% CI, 0.75–1.08; *p* = 0.257).

In the ESCORT-1st trial, 596 Chinese patients with previously untreated advanced or metastatic squamous cell cancer of the esophagus were randomized to camrelizumab (a PD-1 inhibitor) plus paclitaxel and cisplatin or chemotherapy alone [18]. Camrelizumab plus chemotherapy was associated with a significant improvement in median OS of 15.3 months (95% CI, 12.8–17.3) compared to 12.0 months (95% CI, 11.0–13.3) with chemotherapy alone (HR, 0.70; 95% CI, 0.56–0.88; one-sided *p* = 0.0010). Camrelizumab plus chemotherapy was also associated with a better median PFS (6.9 months; 95% CI, 5.8–7.4 vs. 5.6 months; 95% CI, 5.5–5.7; HR, 0.56; 95% CI, 0.46–0.68; one-sided *p* < 0.0001).

Taken together, checkpoint inhibitor immunotherapy plus chemotherapy is a first-line standard option for patients with advanced gastric, esophageal and GEJ cancer. In patients who are not candidates for chemotherapy and have PD-L1-positive tumors (with a CPS ≥ 1), pembrolizumab monotherapy is an appropriate alternate treatment. The benefit of checkpoint inhibitors in patients with PD-L1-negative adenocarcinomas remains inconclusive [31].

#### 5.3.2. Immunotherapy for Previously Treated Patients or Later-Line Therapy

Immune checkpoint inhibitors have demonstrated efficacy in previously treated patients with advanced gastric, esophageal and GEJ adenocarcinomas or squamous cell cancers. Several randomized trials have compared a checkpoint inhibitor alone or in combination with a CTLA-4 inhibitor to chemotherapy or placebo [32,33,34,35,36].

##### Advanced Gastric or GEJ Cancer

In the ATTRACTION-2 phase III trial, 493 Asian patients with advanced gastric or GEJ cancer refractory to ≥ 2 lines of chemotherapy were randomly assigned to nivolumab or placebo [34]. Patients treated with nivolumab had a median OS of 5.26 months compared to 4.14 months with placebo (HR, 0.63; 95% CI, 0.51–0.78; *p* < 0.0001). At 12 months, 26.2% of patients treated with nivolumab were alive compared to 10.9% of patients who received the placebo.

In the CheckMate-032 phase II trial, 160 patients with locally advanced or metastatic chemotherapy-refractory gastric, esophageal or GEJ cancer who previously received ≥2 therapies were randomized to nivolumab 3 mg/kg or nivolumab plus ipilimumab 3 mg/kg and nivolumab 3 mg/kg plus ipilimumab 1 mg/kg [33]. At 12 months, the PFS rates were 8%, 17% and 10%, respectively, whereas the OS rates were 39%, 35% and 24%, respectively.

In the KEYNOTE-061 phase III trial, 592 patients with advanced gastric or GEJ cancer who progressed were randomized to pembrolizumab or paclitaxel [37]. The median OS of patients with tumors with a CPS ≥ 1 who received pembrolizumab was 9.1 months versus 8.3 months with paclitaxel (HR, 0.82; 95% CI, 0.66–1.03; one-sided *p* = 0.0421). The median progression-free survival was 1.5 months with pembrolizumab versus 4.1 months with paclitaxel (HR, 1.27; 95% CI, 1.03–1.57). The updated results of this trial showed a better median OS of 9.1 months versus 8.3 months with pembrolizumab versus chemotherapy in patients with a CPS ≥ 1 (HR, 0.81; 95% CI, 0.66–1.0; *p* = 0.03) and 10.4 months versus 8.3 months in patients with a CPS ≥ 5 (HR, 0.72; 95% CI, 0.53–0.99; *p* = 0.02) [38]. However, the JAVELIN Gastric 300 trial randomized 371 patients with advanced gastric or GEJ cancer who received two lines of therapy with avelumab or physician’s choice of chemotherapy, paclitaxel or irinotecan did not show a survival benefit with checkpoint inhibitors over chemotherapy [32]. The median OS with avelumab was 4.6 months compared to 5.0 months with chemotherapy (HR, 1.1; 95% CI, 0.9–1.4; *p*  =  0.81). The median PFS was 1.4 months versus 2.7 months with avelumab versus chemotherapy (HR, 1.73; 95% CI, 1.4–2.2; *p*  >  0.99).

##### Advanced Esophageal Cancer

In the KEYNOTE-181 trial, 628 previously treated patients with advanced squamous cell or adenocarcinoma of the esophagus were randomized to pembrolizumab or investigator’s choice of chemotherapy (paclitaxel, docetaxel or irinotecan) [36]. In patients with a tumor CPS ≥ 10, pembrolizumab was associated with a median OS of 9.3 months compared to 6.7 months with the investigator’s choice of chemotherapy (HR, 0.69; 95% CI, 0.52–0.93; *p* = 0.0074). The 12-month estimated OS rate was 43% with pembrolizumab versus 20% with chemotherapy. Patients with squamous cell carcinoma who received pembrolizumab had a median OS of 8.2 months compared to 7.1 months with chemotherapy alone (HR, 0.78; 95% CI, 0.63 to 0.96; *p* = 0.0095).

ATTRACTION-3, a phase III trial, randomized 419 patients with previously treated advanced esophageal squamous cell cancer to nivolumab or investigator’s choice of chemotherapy (paclitaxel or docetaxel) [35]. The median OS of patients who received nivolumab was 10.9 months (95% CI, 9.2–13.3) compared to 8.4 months (95% CI, 7.2–9.9) with the chemotherapy group (HR, 0.77; 95% CI, 0.62–0.96; *p* = 0.019).

Based on the data from a randomized trial, immunotherapy should be considered in patients with PD-L1-positive or MSI-H gastric, esophageal and GEJ cancers who have not received an immune checkpoint inhibitor as first-line therapy. The current data suggest limited or no benefit of immunotherapy in patients with low PD-L1-expressing gastric or esophageal adenocarcinoma [31].

## 6. Conclusions

The introduction of immunotherapy in gastric, esophageal and GEJ cancers has changed the treatment landscape. PD-L1 expression is a proposed biomarker to determine the benefit of immunotherapy. A specimen is considered PD-L1 expression positive if CPS is ≥1. In patients with localized esophageal or GEJ cancer who are treated with neoadjuvant chemoradiation therapy and have residual disease following surgery, 1 year of adjuvant nivolumab is recommended to reduce recurrent disease, regardless of PD-L1 status. In patients with previously untreated advanced disease, a checkpoint inhibitor in combination with a platinum based chemotherapy is recommended for PD-L1 positive cancer. Pembrolizumab monotherapy is an appropriate single agent therapy for patients who are not candidates for chemotherapy and have PD-L1-positive tumors with a CPS ≥ 1. Furthermore, immunotherapy should be considered in patients with PD-L1-positive or MSI-H gastric, esophageal and GEJ cancers who have not received an immune checkpoint inhibitor as first-line therapy. Rechallenging with immunotherapy is not recommended if a patient has previously progressed on immunotherapy. The current data suggest limited or no benefit of immunotherapy in patients with low PD-L1-expressing gastric or esophageal adenocarcinoma.

## Figures and Tables

**Table 1:** Summary of pivotal phase III trials evaluating the efficacy of checkpoint inhibitors in patients with previously untreated metastatic gastric, esophageal and GEJ cancers.

Study	Patients	Intervention	Median PFS	Median OS
Keynote-062 [16]	763 patients with advanced gastric or GEJ cancer with PD-L1 CPS score of ≥1	Single agent pembrolizumab or pembrolizumab plus cisplatin and 5FU/cape or chemotherapy alone	2.0 vs. 6.4 months (95% CI, 5.7–7.0) with pembrolizumab vs. chemotherapy; HR, 1.66; 95% CI, 1.37–2.01 in patients with CPS ≥ 1	10.6 vs. 11.1 months; HR 0.91; 99.2% CI, 0.69–1.18 with pembrolizumab vs. chemotherapy in patients with CPS ≥ 1
Checkmate 649 [15]	1581 patients with advanced gastric, esophageal, GEJ adenocarcinoma regardless of PD-L1	Nivolumab plus oxaliplatin and 5FU or cape or nivolumab plus ipilimumab or chemotherapy alone	7.7 vs. 6.05 months; HR 0.68 (98% CI 0.56–0.81) with nivolumab plus chemotherapy vs. chemotherapy in PD-L1 ≥ 5%	14.4 vs. 11.1 months; HR, 0.71 (98.4% CI 0.59–0.86) with nivolumab plus chemotherapy vs. chemotherapy in PD-L1 ≥ 5%
Keynote-590 [17]	749 patients with advanced esophageal or GEJ cancer regardless of PD-L1 status	Pembrolizumab or placebo and 5-FU/cisplatin	6.3 vs. 5.8 months; HR, 0.65 (0.55–0.76) for pembrolizumab plus chemotherapy vs. chemotherapy.	12.4 vs. 9.8 months; HR, 0.73 (0.62–0.86) for pembrolizumab plus chemotherapy vs. chemotherapy alone
Checkmate 648 [13]	970 patients with advanced esophageal squamous cell carcinoma regardless of PD-L1 status	Nivolumab plus 5FU/cisplatin or nivolumab plus ipilimumab or chemotherapy alone	HR, 0.65 (98.5% CI 0.46–0.92) for PFS for nivolumab plus chemotherapy vs. chemotherapy alone in pts with tumor cell PD-L1 ≥ 1%,	15.4 vs. 9.1 months; HR, 0.54 (99.5% CI 0.37–0.80) for nivolumab plus chemotherapy vs. chemotherapy alone in patients with tumor cell PD-L1 ≥ 1%,
Attraction-4 [30]	742 advanced gastric or GEJ cancer	Nivolumab plus chemotherapy (S-1 plus oxaliplatin or CAPOX) or chemotherapy	10.5 vs. 8.3 months with combination vs. chemotherapy alone; (HR 0.68; 98.51% CI 0.51–0.90)	17.5 vs. 17.2 months with combination vs. chemotherapy alone; (HR 0.90; 95% CI 0.75–1.08; *p* = 0.257)
ESCORT-1^st^ [18]	596 patients with advanced squamous cell cancer of esophagus	Camrelizumab plus paclitaxel and cisplatin or chemotherapy alone	6.9 vs. 5.6 months with camrelizumab plus chemotherapy vs. chemotherapy alone; HR, 0.56 (95% CI, 0.46–0.68)	15.3 vs. 12.0 months with camrelizumab plus chemotherapy vs. chemotherapy alone; HR, 0.70 (95% CI, 0.56–0.88)

CPS = Combined positive score; HR = hazard ratio; GEJ = gastroesophageal junction; OS = overall survival; PFS = progression-free survival.

## Supplementary Materials

The following supporting information can be downloaded at: https://www.mdpi.com/article/10.3390/curroncol29050257/s1, Table S1: Western Canadian Gastrointestinal Cancer Conference webinar’s participants list..
