# Supplementary material for: Current Role of Immunotherapy in Gastric, Esophageal and Gastro-Esophageal Junction Cancers—A Report from the Western Canadian Gastrointestinal Cancer Consensus Conference
Source: Curr Oncol. 2022 Apr 29;29(5):3160–70. doi: 10.3390/curroncol29050257 (PMC9139288; doi:10.3390/curroncol29050257)
Supplement: Supplementary file 1 [file curroncol-29-00257-s001.zip › curroncol-1641568-supplementary.pdf]

Supplementary Materials

Table S1. Western Canadian Gastrointestinal Cancer Conference webinar's participants list.

Name	Disciplines	Job Title	Province
Adnan Zaidi	Medical Oncology	Saskatchewan Cancer Agency	SK
Amina Taleb	Medical Oncology	Arnie Charbonneau Cancer Institute	AB
Anahita Dehmoobed	Pharmacy	Cancer Care Manitoba	MB
Bryan Brunet	Radiation Oncology	Saskatchewan Cancer Agency	SK
Chen Zhou	Pathology and Lab Medicine	BC Cancer Agency	BC
Christina Kim	Medical Oncology	CancerCare Manitoba	MB
Corinne Doll	Radiation Oncology	Arnie Charbonneau Cancer Institute	AB
Duc Le	Radiation Oncology	Saskatchewan Cancer Agency	SK
Devin Schellenberg	Radiation Oncology	BC Cancer Agency	BC
Elvira Planincic	Nursing	Cancer Care Manitoba	MB
Emina Torlakovic	Pathology and Lab Medicine	University of Saskatchewan	SK
Henrike Rees	Pathology and Lab Medicine	Saskatchewan Health Authority	SK
Jacob Easaw	Medical Oncology	Cross Cancer Institute	AB
James Paul	Medical Oncology	CancerCare Manitoba	MB
Janine Davies	Medical Oncology	BC Cancer Agency	BC
Jiti Gill	Medical Oncology	BC Cancer Agency	BC
Kamal Haider	Medical Oncology	Saskatchewan Cancer Agency	SK
Karen King	Medical Oncology	Cross Cancer Institute	AB
Karen Mulder	Medical Oncology	Cross Cancer Institute	AB
Keith Tankel	Radiation Oncology	Cross Cancer Institute	AB
Kelly Cheung	Pharmacy	CancerCare Manitoba	MB
Kimberly Hagel	Medical Oncology	Saskatchewan Cancer Agency	SK
Lori Walker	Nursing	Cancer Care Manitoba	MB
Louise Quenneville	Pathology and Lab Medicine	Saskatchewan Health Authority	SK
Maria Augusta Safro	Medical Oncology	BC Cancer Agency	BC
Mark Kristjanson	Community Oncology	CancerCare Manitoba	MB
Michael Lee	Medical Oncology	Peter MacCallum Cancer Centre	AUS
Mussawar Iqbal	Medical Oncology	Saskatchewan Cancer Agency	SK
Omar Abdelsalam	Medical Oncology	Cross Cancer Institute	AB
Osama Ahmed	Medical Oncology	Saskatchewan Cancer Agency	SK
Rani Kanthan	Pathology and Lab Medicine	University of Saskatchewan	SK
Rebekah Rittberg	Medical Oncology	CancerCare Manitoba	MB
Sangjune Laurence Lee	Radiation Oncology	Arnie Charbonneau Cancer Institute	AB
Shahid Ahmed	Medical Oncology	Saskatchewan Cancer Agency	SK
Sharlene Gill	Medical Oncology	BC Cancer Agency	BC
Shaun Loewen	Radiation Oncology	Arnie Charbonneau Cancer Institute	AB
Sheryl Koski	Medical Oncology	Cross Cancer Institute	AB
Simon Yu	Medical Oncology	Burnaby Hospital Cancer Centre	BC
Stephanie Lelond	Nursing	CancerCare Manitoba	MB
Tehmina Asif	Medical Oncology	BC Cancer Agency	BC
Thuan Do	Medical Oncology	BC Cancer Agency - Surrey	BC
Tirath Nijjar	Radiation Oncology	Cross Cancer Institute	AB
Vallerie Gordon	Medical Oncology	CancerCare Manitoba	MB
Vincent Tam	Medical Oncology	Arnie Charbonneau Cancer Institute	AB
Wei Ning Jiang	Radiation Oncology	BC Cancer Agency	BC
Wei Xiong	Pathology and Lab Medicine	University of British Columbia	BC

William Hunter	Radiation Oncology	CancerCare Manitoba	MB
Deepti Ravi	Pathology and Lab Medicine	Saskatchewan Health Authority	SK
Howard Lim	Medical Oncology	BC Cancer Agency	BC
Teresa Tiano	Chair & Co-Founder	My Gut Feeling - Stomach Cancer Foundation of Canada	ON
